# Validity and reliability of a cognitive function assessment tool using psychomotor function tests in patients with alcohol use disorders – a pilot study –

**DOI:** 10.1186/s40780-026-00584-0

**Published:** 2026-05-16

**Authors:** Yuma Shimizu, Junichi Miyaura, Manako Hanya, Masahide Okuda, Hiroaki Mizutani, Hiroyuki Kamei

**Affiliations:** 1 Office of Clinical Pharmacy Practice and Health Care Management, Graduate School of Pharmacy, Meijo University, 150 Yagotoyama, Tenpaku-ku, Nagoya city, Aichi 468-8503 Japan; 2 Office of Clinical Pharmacy Practice and Health Care Management, Faculty of Pharmacy, Meijo University, Nagoya, Aichi Japan; 3 Department of Psychiatry, Yagoto Hospital, Nagoya, Aichi Japan

**Keywords:** Alcohol use disorder, Cognitive function, Cognitive function assessment, Psychomotor function test

## Abstract

**Background:**

Even after alcohol detoxification, 50–80% of patients with alcohol use disorder (AUD) exhibit cognitive impairments, which affect key domains of social functioning, such as employment, interpersonal relationships, and communication. Therefore, it is necessary to assess cognitive function in patients with AUD in routine practice. However, existing cognitive function assessments are difficult to apply in routine practice because they require a long time, are burdensome on patients, and are limited to evaluators with special training. The present study investigated the relationship between a simple and novel assessment, called Psychomotor Function Tests (PFT), and existing Neuropsychological Tests (NT) in patients with AUD.

**Methods:**

Twenty patients with AUD underwent a cognitive assessment using NT (Raven’s Colored Progressive Matrices, Frontal Assessment Battery (FAB), and Clinical Assessment for Attention (CAT)) as well as tablet-based PFT, with each assessment being conducted twice: 1 week and 2 months after alcohol detoxification with benzodiazepines. Twenty healthy controls underwent the same assessment.

**Results:**

Psychomotor function assessed by PFT was significantly worse in patients with AUD than in healthy controls (*p* < 0.05). At both assessment time points, PFT composite score correlated with the total FAB score (*r* = 0.500, 0.458) and CAT time required (*r* = -0.447, -0.496). The CRT, CTT-TT, CTT-PAT and RVIP demonstrated a degree of test-retest reliability (*r* = 0.816, 0.812, 0.797, 0.676).

**Conclusions:**

The correlations observed between PFT and conventional NT suggest the utility of PFT for evaluating cognitive function in patients with AUD in clinical settings.

## Introduction

Excessive alcohol consumption is a risk factor for several physical diseases, such as lifestyle-related diseases and cancer, as well as psychiatric disorders, including depression and bipolar disorder [1]. Approximately 3.4% of the population in Japan meet the diagnostic criteria for alcohol use disorder (AUD) [2], which is a pressing public health issue. AUD is a chronic disease with a high relapse rate [3]. A previous study demonstrated that cognitive impairments were present in approximately 36.1% of patients in acute hospital settings and 66.0-68.8% of patients in addiction treatment units [4]. Even after alcohol detoxification, cognitive impairments persisted in 50–80% of patients with AUD [5, 6]. Many cognitive domains are impaired in patients with AUD, such as attention and working memory [7], and contribute to difficulties with social functioning, including employment, interpersonal relationships, and communication [8]. Cognitive impairments in AUD are also associated with poor treatment adherence [9] and treatment outcomes [10]. Therefore, it is important to focus on cognitive impairments in AUD in clinical settings. The frontal lobe has been implicated in behavioral dysfunctions resulting from long-term alcohol use, such as impulsivity, cognitive/behavioral flexibility, and attention deficits [11].

The assessment of cognitive function in patients requires the use of a combination of several neuropsychological tests (NT), which are time-consuming and burdensome for both the patient and evaluator [12]. Furthermore, these tests often require specialized training, which limits their applicability in clinical practice.

Based on these backgrounds, we have conducted psychomotor function tests (PFT) using specialized equipment for patients with schizophrenia, enabling the evaluation of cognitive function, within 20 min [13]. PFT comprises four tasks: Critical Flicker Fusion (CFF), Choice Reaction Time (CRT), Compensatory Tracking Task (CTT), and Rapid Visual Information Processing (RVIP). These tasks mainly evaluate attention and working memory. Furthermore, we developed a tablet-based version of PFT, which is more easily applicable in daily clinical settings. Tablet-based PFT showed correlations with the Trail Making Test, which assesses general attention, working memory, and processing speed in patients with schizophrenia. Therefore, tablet-based PFT were confirmed to be a valid method for assessing cognitive function in patients with schizophrenia [14].

In the present study, we expanded the application of tablet-based PFT to patients with AUD and examined its validity and reliability as a cognitive function assessment tool.

## Methods

### Subjects

The present study included 20 inpatients aged 20 years or older who were diagnosed based on the Diagnostic and Statistical Manual of Mental Disorders (DSM) - Ⅴ criteria for AUD at Yagoto Hospital between January 2023 and January 2025. Inclusion criteria were as follows: (1) the absence of diseases that affect psychomotor function other than AUD, (2) the absence of a history of drug or substance abuse other than alcohol, and (3) a regular sleep/wake cycle that does not affect psychomotor function. Participants were clinically stable in terms of having no alcohol withdrawal symptoms.

Twenty healthy controls (HCs) aged 20 years or older who met the following criteria were included: (1) Alcohol Use Disorders Identification Test (AUDIT) score < 8 [15], (2) a Mini-Mental State Examination score ≥ 24 [16, 17], (3) a Raven’s Colored Progressive Matrices (RCPM) score ≥ 24 [18], (4) no history of diseases affecting psychomotor function, (5) no use of medications that affect psychomotor function, (6) no history of drug or substance abuse, and (7) a regular sleep/wake cycle that does not affect psychomotor function. Based on a previous study [14], inclusion and exclusion criteria were established. In addition, eligibility for inclusion in the HC group was confirmed based on the final medical judgment of a physician. HCs were recruited through announcements to the general public at Meijo University and Yagoto Hospital.

Following hospitalization, all patients were prohibited from alcohol consumption, having passed a period of at least three weeks between their final alcohol consumption and the first cognitive assessment. Furthermore, all patients were receiving treatment with benzodiazepines for the detoxification of alcohol. Cognitive assessments were conducted on HCs after the confirmation of no alcohol consumption on the day of the study.

### Study procedures

#### Assessment procedure

**Fig. 1:**
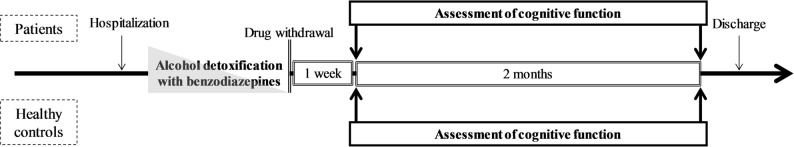
Flowchart of study procedures

Figure 1 shows the flow of the study. We administered PFT using a tablet device (nonoi Co., Ltd.), as well as NT combining RCPM [18, 19], Frontal Assessment Battery (FAB) [20, 21], and Clinical Assessment for Attention (CAT) [22]. We used the Clinical Global Impressions (CGI) scale [23] to examine the severity of AUD. PFT was conducted by pharmacists and NT and CGI by physicians. PFT, NT, and CGI were evaluated at the same time.

PFT, RCPM, and FAB were performed by patients and HCs using the same procedure. CAT, for which standardized HC data are available [24], and CGI were performed exclusively by the patient group.

All patients abstained from alcohol after admission and were treated with benzodiazepines to prevent withdrawal symptoms. In accordance with a previous study [25], the first cognitive assessment was conducted one week after detoxification to eliminate any effect of benzodiazepines on cognitive function. The second assessment was performed two months after the first assessment. HCs underwent cognitive assessments on a similar schedule.

#### Assessment scales

##### Psychomotor function tests (PFT)

Psychomotor function was evaluated using a test battery consisting of the following four tasks [13, 14, 26, 27]. All tasks were performed with the tablet placed on the desk, and the illuminance on the tablet was the same for all participants.

##### Critical flicker fusion (CFF)

CFF evaluates the integrative capacity of the central nervous system, specifically sensory ability to discern the intermittent flicking of light [14]. A lower threshold indicates a decrease in the overall activity of the central nervous system. The test set-up on the tablet screen consisted of four red lights arranged in a 1-cm square. Lights flicked on and off at a constantly increasing or decreasing rate. Subjects were required to discriminate flicker from fusion and vice versa. The CFF threshold (Hz) for each subject was selected by averaging eight measurements: four ascending (flicker to fusion) and four descending (fusion to flicker) measurements.

##### Choice reaction time (CRT)

CRT is used as an indicator of sensorimotor ability, and assesses the ability to focus on and respond to a visual stimulus [14]. In CRT, a higher score indicates a decline in sensorimotor function. The tablet screen consisted of a start button in the middle as well as 6 reaction buttons aligned in a fan shape at equal intervals. Each reaction button had red and green lights that were also aligned in a fan shape, and the green light turned on prior to the red light. After placing the index finger on the start button, subjects were required to use the green light as a warning to react quickly when the red light turned on. Subjects were required to press the reaction button next to the red light as soon as it lit up in order to turn it off. The number of warning green lights gradually increased (1, 3, and 6 lights in this order) and decreased (6, 3, and then 1 light). The time between the red light coming on and the finger pushing the action button was taken as CRT.

##### Compensatory tracking test (CTT)

CTT consists of the following dual tasks: a tracking task (TT) and peripheral awareness task (PAT), which subjects were required to perform simultaneously.

##### Tracking task (TT)

TT assesses the ability to react to and track an object [14]. In TT, a higher score indicates an impaired ability to accurately track an object. Subjects were required to track a round object that appeared on the tablet screen. The object moved along a straight line in a random manner, and subjects were asked to place the cursor in the middle of the object using a wireless mouse and track its movement. The distance between the center of the object and the cursor was measured in pixels, and performance was assessed as the mean distance between the two points for samples randomly selected during the 9-minute test.

##### Peripheral awareness task (PAT)

PAT was performed at the same time as TT, ensuring that it did not interfere with TT [14]. A higher score in PAT indicates an impaired ability to divide attention. PAT assesses peripheral awareness and lasts for 9 min, similar to TT. In PAT, 50 stimuli (yellow objects) randomly appeared at the 4 corners of the tablet screen, and subjects were required to push the reaction button as soon as they saw the yellow object while performing TT. The average time interval between the appearance of the object and pushing the reaction button was calculated in msec.

##### Rapid visual information processing (RVIP)

RVIP was used as a means to assess working memory, sustained attention, and concentration on cyclical events [14]. A higher score in RVIP indicates better sustained attention and concentration. Subjects were required to monitor a series of single digits (0–9) appearing on the screen at a rate of 100 digits every minute and respond to consecutive sequences of three odd or even digits using a wireless mouse button during the 9-min test period. The assessment measured the rate of correct responses.

##### Neuropsychological tests (NT)

The following three NT, which are routinely used in daily clinical practice, were performed. Since patients with AUD exhibit impairments in frontal lobe functions, such as attention [11, 28, 29], we assessed their cognitive function using a combination of FAB [20, 21] and CAT [22], the former of which allows for an easy and comprehensive evaluation of frontal lobe function, while the latter assesses attention. Additionally, RCPM [18, 19] was combined to assess intellectual ability. Since these three tests have been reported as a cognitive function assessment for patients with AUD in Japan [30], the present study also employed the three NT as cognitive function tests. The NT was administered by a clinical psychologist or speech–language therapist trained in neuropsychological assessment and required approximately 30–40 min to complete the three tasks.

##### Raven’s colored progressive matrices (RCPM)

In RCPM, subjects were required to select only one of the six choice designs that matched the missing part of the design [18, 19]. There was no time limit. The number of correct answers out of 36 standard designs was assessed as intellectual ability.

##### Frontal assessment battery (FAB)

FAB was conducted in the form of questions and associated with a number of functions, such as attention, concept change (retention and response inhibition), and fluency [20, 21]. In FAB, six tasks were conducted: (1) a conceptualization task, (2) mental flexibility task, (3) motor programming task, (4) sensitivity to interference task, (5) inhibitory control task (Go/No Go task), and (6) environmental autonomy task, and the total score out of 18 was assessed.

##### Clinical assessment for attention (CAT)

In CAT, the visual cancellation task was conducted. Subjects were required to choose the designated objects in three modalities: shapes, numbers, and letters [22]. In the present study, we assessed the total time required (sec) for each modality. Healthy normative data are available by purchasing CAT.

#### Clinical global impressions (CGI)

The severity of AUD was evaluated by CGI-Severity (CGI-S), ranging from 1 (normal) to 7 (very severe), with a higher score indicating more severe symptoms [23].

### Statistical analysis

Using the statistical analysis software IBM SPSS ver. 29.0 (IBM Japan, Tokyo), Spearman’s rank correlation coefficient test was performed to examine the relationships between assessment scales. Intraclass correlation coefficients were used to confirm test-retest reliability based on HC data. Normality was assessed using the Shapiro–Wilk test because of the small sample size. Parametric tests were used for normally distributed data, whereas nonparametric tests were applied when the assumption of normality was not met. In addition, the Mann-Whitney test and *χ*^2^ test were used to compare scores and background factors between groups, and the paired *t*-test in the case of a normal distribution and the Wilcoxon signed-rank test to compare within groups. The significance level was set at *p* < 0.05. The degree of cognitive dysfunction in each patient was evaluated by calculating the *Z*-score [31]. *Z*-scores were calculated to indicate a reduced ability when the *Z*-score decreased. In PFT, the average of the five *Z*-scores was assessed as a composite score.

In psychology, correlation coefficients are generally interpreted as follows: 0.1–0.3, weak; 0.4–0.6, moderate; 0.7–0.9, strong; and 1.0, perfect [32]. Accordingly, correlation coefficients of 0.4 or higher were considered to indicate a meaningful degree of correlation.

The present study employed tablet-based PFT reported in the previous study [14], but: (1) the population of the present study differed from the previous study [14], as it comprised patients with alcohol use disorder at Yagoto Hospital. (2) The present study examined correlations with different neuropsychological tests (RCPM, FAB, CAT) than those in the previous study [14] and verified validity. (3) While the previous study involved a single assessment point, the present study employed two assessment points over 8 months period. (4) The present study verified test-retest reliability, which was not examined in the previous study [14]. Consequently, as the present study differs entirely from the previous study in terms of the implementing facility, population (disorder), neuropsychological tests, assessment time points, and purpose of analysis, the present study was reported as a novel study.

## Results

### Clinical characteristics

Eighty-nine patients with AUD and 22 HCs were screened. Sixty-three patients and 2 HCs who did not meet the eligibility criteria were excluded. Additionally, 4 patients dropped out and 2 declined to participate, leaving 20 patients and 20 HCs in the final analysis. After the removal of participants who met the exclusion criteria, the final sample of 20 patients and 20 HCs was included in the analysis.

Table 1 shows the baseline clinical characteristics of the 20 patients and 20 HCs. Patients had a short duration of disease and a slightly higher age. No significant differences were observed in age or sex between the groups, while the duration of education was significantly shorter for patients than for HCs. In addition, alcohol consumption at hospitalization and the AUDIT score were significantly higher for patients than for HCs, indicating problems with alcohol use.

**Table 1:** Demographic data of subjects

Variable		Patients (*n* = 20)	Healthy controls (*n* = 20)	*p* value
Age (years)		53.0 [47.3–63.0]	50.0 [40.3–55.0]	0.056^a^
Sex	Male	15 (75.0%)	14 (70.0%)	0.723^b^
	Female	5 (25.0%)	6 (30.0%)	
Duration of education (years)		14.0 [12.0–16.0]	16.0 [16.0–18.0]	< 0.001^a^
Duration of illness (years)		0.0 [0.0–0.0]	―	―
Age at beginning of alcohol consumption (years)		20.0 [18.0–20.0]	20.0 [20.0–20.0]	0.072^a^
Alcohol consumption (g/day)		134.5 [57.5–174.0]	0.0 [0.0–5.0]	< 0.001^a^
AUDIT score		26.5 [22.3–29.8]	2.5 [1.0–4.0]	< 0.001^a^
CGI-S score		3.0 [3.0–3.0]	―	―

a: Mann-Whitney U test, b: *χ*^2^ test

AUDIT: Alcohol Use Disorders Identification Test, CGI-S: Clinical Global Impressions-Severity

### Scores of cognitive functions

#### Comparison of cognitive functions between groups

As shown in Table 2–1, the results of cognitive assessments were compared between patients with AUD and HCs. Patients had significantly worse psychomotor function in PFT, except CTT-PAT, than HCs. CTT-PAT was slightly worse in patients than in HCs (*p* = 0.076). No significant differences were observed in NT between the groups.

**Table 2-1-1:** Comparisons of scores of psychomotor function tests between groups

Assessment items		Patients(n = 20)	Healthy controls(n = 20)	p value^a^	Z-score	
1st time (1 week after alcohol detoxification)						
CFF (Hz)		36.1 [30.7-39.4]	41.0 [36.4-44.1]	0.009	-0.85[-1.96, -0.18]	
CRT (msec)		1094.1 [904.1-1189.9]	904.7 [809.7-982.5]	0.004	-1.19[-2.27, -0.08]	
CTT	TT (pixel)	29.1 [26.3-40.4]	20.1 [17.4-23.4]	<0.001	-1.80[-4.32, -1.18]	
	PAT (msec)	596.1 [551.8-703.0]	550.3 [468.1-646.6]	0.076	-0.12[-0.88, 0.19]	
RVIP (%)		47.6 [28.8-58.5]	57.5 [50.9-75.0]	0.014	-0.86[-1.95, -0.24]	
Composite score					-1.01[-1.49, -0.70]	
2nd time (2 months after alcohol detoxification)						
CFF (Hz)		35.7 [34.2-38.6]	39.7 [35.7-41.9]	0.052	-0.95 [-1.25, -0.35]	
CRT (msec)		979.3 [897.9-1152.9]	875.4 [792.9-926.3]	0.002	-0.66 [-1.99, -0.36]	
CTT	TT (pixel)	28.7 [22.8-33.5]	20.1 [17.1-23.3]	<0.001	-1.72 [-2.78, -0.41]	
	PAT (msec)	584.0 [539.8-721.9]	520.0 [454.9-631.5]	0.038	-0.04 [-1.01, 0.27]	
RVIP (%)		52.8 [37.3-61.8]	66.0 [60.3-87.2]	0.009	-0.56 [-1.45, -0.05]	
Composite score					-1.08 [-1.43, -0.17]	

CFF: Critical Flicker Fusion, CRT: Choice Reaction Time, CTT: Compensatory Tracking Test, TT: Tracking Task, PAT: Peripheral Awareness Task, PVIP: Rapid Visual Information Processing, RCPM: Raven’s Colored Progressive Matrices, Frontal Assessment Battery: FAB, Clinical Assessment for Attention: CAT

**Table 2-1-2:** Comparisons of scores of neuropsychological tests between groups

Assessment items		Patients(n = 20)	Healthy controls(n = 20)	p value^a^	Z-score
1st time (1 week after alcohol detoxification)					
RCPM	total score	32.0 [31.0-35.0]	34.0 [32.3-35.0]	0.620	―
FAB	total score	17.0 [16.3-18.0]	17.0 [17.0-18.0]	0.779	―
CAT	time required (sec)	280.5 [259.5-339.5]	―	―	-0.69 [-1.21, 0.16]
2nd time (2 months after alcohol detoxification)					
RCPM	total score	33.0 [31.3-35.0]	34.0 [31.3-35.0]	0.192	―
FAB	total score	17.0 [17.0-18.0]	17.5 [17.0-18.0]	0.883	―
CAT	time required (sec)	272.5 [259.5-316.8]	―	―	-0.48 [-0.92, 0.02]

a: Mann-Whitney U testCFF: Critical Flicker Fusion, CRT: Choice Reaction Time, CTT: Compensatory Tracking Test, TT: Tracking Task, PAT: Peripheral Awareness Task, PVIP: Rapid Visual Information Processing, RCPM: Raven’s Colored Progressive Matrices, Frontal Assessment Battery: FAB, Clinical Assessment for Attention: CAT

In the second assessment, patients had significantly worse psychomotor function in PFT, except CFF, than HCs. CFF was slightly worse in patients than in HCs (*p* = 0.052). No significant differences were observed in NT between the groups.

**Table 2-2-1:** Comparisons of cognitive function tests in patients with alcohol use disorder

Assessment items		1st time	2nd time	p value ^a^
Psychomotor Function Tests				
CFF (Hz)		36.1 [30.7-39.4]	35.7 [34.2-38.6]	0.614
CRT (msec)		1094.1 [904.1-1189.9]	979.3 [897.9-1152.9]	0.232
CTT	TT (pixel)	29.1 [26.3-40.4]	28.7 [22.8-33.5]	0.021
	PAT (msec)	596.1 [551.8-703.0]	584.0 [539.8-721.9]	0.332
RVIP (%)		47.6 [28.8-58.5]	52.8 [37.3-61.8]	0.022
Neuropsychological Tests				
RCPM	total score	32.0 [31.0-35.0]	33.0 [31.3-35.0]	0.580
FAB	time required (sec)	17.0 [16.3-18.0]	17.0 [17.0-18.0]	0.589
CAT	total score	280.5 [259.5-339.5]	272.5 [259.5-316.8]	0.059

CFF: Critical Flicker Fusion, CRT: Choice Reaction Time, CTT: Compensatory Tracking Test, TT: Tracking Task, PAT: Peripheral Awareness Task, PVIP: Rapid Visual Information Processing, RCPM: Raven’s Colored Progressive Matrices, Frontal Assessment Battery: FAB, Clinical Assessment for Attention: CAT

#### Comparisons of cognitive function within groups

Within-group comparisons of cognitive assessments at each time point are shown in Table < link rid="tb8”>2–2. Among patients, no significant changes were noted in NT, while CTT-TT scores in PFT were significantly lower in the second assessment than in the first. In addition, scores in RVIP were significantly higher in the second assessment than in the first. In HCs, no significant differences were observed in any cognitive function assessments between the two time points. The intraclass correlation coefficients of HC data were low at 0.407 for CFF, but high at 0.816 for CRT, 0.812 for CTT-TT, 0.797 for CTT-PAT, and 0.676 for RVIP.

**Table 2-2-2:** Comparisons of cognitive function tests in healthy controls

Assessment items		1st time	2nd time	p value
Psychomotor Function Tests				
CFF (Hz)		40.3 ± 4.8	38.8 ± 4.3	0.205^a^
		(Correlation coefficient: 0.407)		0.033^b^
CRT (msec)		893.2 ± 130.5	862.3 ± 123.7	0.089^a^
		(Correlation coefficient: 0.816)		<0.001^b^
CTT	TT (pixel)	21.0 ± 4.5	21.1 ± 4.9	0.936^a^
		(Correlation coefficient: 0.812)		<0.001^b^
	PAT (sec)	578.6 ± 141.7	543.7 ± 114.1	0.072^a^
		(Correlation coefficient: 0.797)		<0.001^b^
RVIP (%)		62.6 ± 17.4	68.5 ± 19.7	0.095^a^
		(Correlation coefficient: 0.676)		<0.001^b^
Neuropsychological Tests				
RCPM	total score	34.0 [32.3-35.0]	34.0 [31.3-35.0]	0.399^c^
FAB	time required (sec)	17.0 [17.0-18.0]	17.5 [17.0-18.0]	0.506^c^

a: paired t-test, b: Intraclass Correlation Coefficient, c: Wilcoxon signed-rank testCFF: Critical Flicker Fusion, CRT: Choice Reaction Time, CTT: Compensatory Tracking Test, TT: Tracking Task, PAT: Peripheral Awareness Task, PVIP: Rapid Visual Information Processing, RCPM: Raven’s Colored Progressive Matrices, Frontal Assessment Battery: FAB

### Relationships between Psychomotor Function Tests (PFT) and Neuropsychological Tests (NT) in patients

The relationships between PFT and NT in patients are shown in Table 3. In the first assessment, the total score of FAB correlated with *Z*-scores in CRT and CTT-TT and the composite score (*r* = 0.465, *r* = 0.532, and *r* = 0.500, respectively). The composite score also correlated with the time required for CAT (*r* = -0.447).

**Table 3:** Correlation coefficients of cognitive function tests in patients with alcohol use disorder

			Psychomotor function tests (*Z*-score)					
			CFF	CRT	CTT- TT	CTT- PAT	RVIP	Composite score
1st time								
Neuropsychological Tests	RCPM	total score	-0.042	-0.052	0.192	0.351	0.287	0.188
	FAB	total score	-0.194	0.465*	0.532*	0.262	0.373	0.500*
	CAT	time required (sec)	-0.197	-0.350	-0.406	-0.432	-0.074	-0.447*
2nd time								
Neuropsychological Tests	RCPM	total score	-0.114	0.084	0.298	0.295	0.404	0.326
	FAB	total score	0.182	0.286	0.485*	0.378	0.470*	0.458*
	CAT	time required (sec)	-0.512*	-0.447*	-0.403	-0.365	-0.116	-0.496*

Spearman’s rank correlation coefficient [**p < 0.01, *p < 0.05) [*n* = 20)

CFF: Critical Flicker Fusion, CRT: Choice Reaction Time, CTT: Compensatory Tracking Test, TT: Tracking Task, PAT: Peripheral Awareness Task, PVIP: Rapid Visual Information Processing, RCPM: Raven’s Colored Progressive Matrices, Frontal Assessment Battery: FAB, Clinical Assessment for Attention: CAT

In the second assessment, the total score of FAB correlated with the *Z*-scores of CTT-TT and RVIP and the composite score (*r* = 0.485, *r* = 0.470, and *r* = 0.458, respectively). The time required for CAT also correlated with the *Z*-scores of CFF and CRT and the composite score, respectively (*r* = -0.512, *r* = -0.447, and *r* = -0.496, respectively).

## Discussion

Cognitive impairments in patients with AUD have been identified as barriers to social functioning; therefore, the development of a simple, objective cognitive assessment tool is needed. The present study revealed correlations between our newly developed PFT and conventional NT, and confirmed the validity of the new assessment.

In this study, no significant differences were observed in any NT scores between the groups. A previous study reported that RCPM scores were significantly lower in patients with AUD than in HCs [25]. RCPM scores in the present study were not consistent with these findings, which may have been due to the different inclusion criteria and sample sizes. Additionally, previous studies reported that the FAB score was significantly lower in patients with AUD than in HCs [33, 34], which is inconsistent with the results obtained herein. Since FAB is a screening tool for the detection of frontal lobe dysfunction [35], it may be difficult to precisely evaluate the degree of cognitive impairment associated with AUD. Furthermore, the *Z*-score of CAT in the present study was better than the *Z*-score of attention assessed by the Brief Assessment of Cognition in Schizophrenia Japanese Version (BACS-J) in a previous study [36]. Although the criteria in the present study may have been strict and the number of patients may have been limited, difficulties are associated with fully assessing the degree of cognitive impairment using the three NT tasks only.

In the present study, PFT revealed that performance in multiple tasks was significantly lower in patients with AUD than in HCs. These results are consistent with previous studies that compared psychomotor function in patients with AUD and HCs using a single task in each study [37, 38]. In the present study, the composite score of PFT was about − 1.0, which were similar to the composite score of cognitive function reported in previous studies using tools such as the Brief Examination of Alcohol-Related Neuropsychological Impairment and BACS-J [36, 39]. In the present study, the specific PFT items that showed significant correlations differed between the first and second assessments. This may be attributable to changes in the clinical condition of hospitalized patients over time, as well as to the limited sample size. However, the PFT composite score consistently correlated with CAT completion time and the total FAB score at both assessment points. These findings suggest that the PFT may be a useful tool for assessing cognitive function in patients with AUD. Additionally, there were no significant differences in the results of PFT between the first and second assessments among HCs, indicating the absence of a practice effect over at least a two-month interval. Furthermore, although the reliability coefficient for CFF was low at 0.441, those for CRT and CTT-TT surpassed 0.81 and were assessed as ‘almost perfect’ reliability, and that for CTT-PAT and RVIP were evaluated as ‘substantial’ at 0.61 or above, indicating high test-retest reliability.

While previous studies focused on psychomotor function in patients with AUD [38], few studies have examined composite batteries combining multiple psychomotor tasks. PFT used in the present study, comprising four tasks, allowed for a comprehensive assessment of psychomotor function. Despite this combination, the total testing time was approximately 20 min, making it relatively quick and minimally burdensome for both patients and evaluators. The use of tablet-based PFT enabled usability, while the gamified nature of the test made it more accessible and engaging for patients. Furthermore, since all assessments of PFT are conducted using tablet devices, evaluators require no special training or assessment, making it useful for evaluating cognitive function in patients with AUD in the real world of clinical practice. The present study demonstrated the potential of PFT to assess frontal lobe function, including attentional function, in patients with AUD, suggesting prospects for its clinical implementation in the near future.

There are several limitations that need to be addressed. The sample consisted of patients who were capable of completing PFT and did not have comorbid psychiatric disorders, such as major depression or bipolar disorder. Furthermore, the patients in this study were slightly older and had a shorter duration of disease. Because the patients in the present study had a relatively short duration of AUD and mild disease severity, conventional neuropsychological tests may not have detected significant group differences. Therefore, the generalization of these results for direct comparisons with the findings of other studies may be difficult. Furthermore, although this study focused on cognitive function, the ultimate treatment goal for AUD is social recovery. Further research needs to be performed with a focus on social participation, including employment. Finally, since this was a pilot study, the number of cases and cognitive domains were limited and, thus, further research is needed. The correlations between the PFT score and the FAB/CAT scores observed in this study were of moderate magnitude, suggesting a certain degree of convergent validity. However, further investigation is needed to determine their clinical usefulness. However, there is some value in using PFT clinically as a supplement to a cognitive function assessment, and the use of PFTs for evaluating cognitive function is expected in the future.

## Conclusion

In patients with AUD, the results of tablet-based PFT correlated with those of NT, suggesting its utility as a novel cognitive assessment tool.

## Data Availability

No datasets were generated or analysed during the current study.
